# The High Level of Aberrant Splicing of *ISCU* in Slow-Twitch Muscle May Involve the Splicing Factor SRSF3

**DOI:** 10.1371/journal.pone.0165453

**Published:** 2016-10-26

**Authors:** Denise F. R. Rawcliffe, Lennart Österman, Hans Lindsten, Monica Holmberg

**Affiliations:** 1 Department of Medical Biosciences, Medical and Clinical Genetics, Umeå University, Umeå, Sweden; 2 Department of Pharmacology and Clinical Neuroscience, Clinical Neuroscience, Umeå University Hospital, Umeå, Sweden; University of Minnesota Medical Center, UNITED STATES

## Abstract

Hereditary myopathy with lactic acidosis (HML) is an autosomal recessive disease caused by an intronic one-base mutation in the *iron-sulfur cluster assembly* (*ISCU*) gene, resulting in aberrant splicing. The incorrectly spliced transcripts contain a 100 or 86 bp intron sequence encoding a non-functional ISCU protein, which leads to defects in several Fe-S containing proteins in the respiratory chain and the TCA cycle. The symptoms in HML are restricted to skeletal muscle, and it has been proposed that this effect is due to higher levels of incorrectly spliced *ISCU* in skeletal muscle compared with other energy-demanding tissues. In this study, we confirm that skeletal muscle contains the highest levels of incorrect *ISCU* splice variants compared with heart, brain, liver and kidney using a transgenic mouse model expressing human HML mutated *ISCU*. We also show that incorrect splicing occurs to a significantly higher extent in the slow-twitch soleus muscle compared with the gastrocnemius and quadriceps. The splicing factor serine/arginine-rich splicing factor 3 (SRSF3) was identified as a potential candidate for the slow fiber specific regulation of *ISCU* splicing since this factor was expressed at higher levels in the soleus compared to the gastrocnemius and quadriceps. We identified an interaction between SRSF3 and the *ISCU* transcript, and by overexpressing SRSF3 in human myoblasts we observed increased levels of incorrectly spliced *ISCU*, while knockdown of SRSF3 resulted in decreased levels. We therefore suggest that SRSF3 may participate in the regulation of the incorrect splicing of mutant *ISCU* and may, at least partially, explain the muscle-specific symptoms of HML.

## Introduction

Patients suffering from the autosomal recessive disease hereditary myopathy with lactic acidosis (HML) were first described in 1964 by Larsson et al. [1]. The patients displayed poor physical performance and a decreased tolerance to exercise, in which even low-level exercise resulted in symptoms such as palpitations, dyspnea, muscle cramps, and tachycardia as well as an increased release of pyruvate and lactate. Extensive exercise and extreme diets like fasting can induce severe episodes of the disease, characterized by severe acidosis and myoglobinuria, to such an extent that it can be fatal [1, 2]. Biochemical studies of the skeletal muscle of patients with HML have shown decreased levels and activity of several iron-sulfur (Fe-S)-containing proteins in the mitochondrial respiratory chain and the tricarboxylic acid (TCA) cycle, including mitochondrial aconitase and complex I, II (succinate dehydrogenase; SDH) and III [2–7]. However, no obvious abnormalities of the patient’s heart function or central nervous system have been found [1]. Combined, these observations suggest pathological muscle metabolism in HML patients.

The disease has been shown to be caused by an intronic mutation in the iron-sulfur cluster assembly gene, *ISCU* [5, 8, 9]. The ISCU protein functions as a scaffold protein in the formation of Fe-S clusters, which are present in numerous proteins involved in a wide range of cellular processes [10]. The ISCU protein has been shown to be essential for survival in numerous species and a complete knock-out of *ISCU* in mice lead to early embryonic death [7]. The intronic mutation identified in the HML patients is a one base-pair substitution (G→C) located 382 bp downstream of exon 4 (g. 7044 G>C). The mutation activates cryptic splice sites, where the acceptor site is located 6 bp downstream of the mutation [5, 8, 9]. This leads to aberrant splicing of the *ISCU* gene, where 100 bp of intron 4 is included in the final mRNA transcript [5, 8, 9]. An alternative inclusion of 86 bp also exists, in which the same acceptor splice site is coupled with an earlier donor splice site [9]. We have previously shown that the aberrant splicing also occurs in individuals not carrying the disease-specific mutation but to a much lower extent in all examined tissues [7]. The introduced pseudoexon in the *ISCU* mRNA results in 15 novel amino acids followed by a premature stop codon disrupting the last α-helix of the ISCU protein [5, 8, 9].

In HML patients, incorrect splicing is much more prominent in skeletal muscle compared to heart and liver tissue [7]. Mutant *ISCU* mRNA carrying the pseudoexon has been shown to represent almost 80% of the total *ISCU* mRNA in patient muscle tissue, compared to 46% for liver tissue and only 30% for heart tissue [7]. We, and others, have previously shown that muscle from HML patients shows decreased levels of ISCU protein, which correlated well with the high level of incorrect splicing in muscle [5, 7]. The tissue-specific incorrect splicing of *ISCU*, resulting in decreased levels of ISCU protein and abnormal Fe-S cluster formation, might thereby explain the muscle-specific phenotype in HML patients.

As splicing is a highly complex regulatory process, the splicing of *ISCU* most likely involves a collection of splicing factors where each participant plays a specific role. We have previously identified PTBP1, IGF2BP1 and RBM39 as modulators of the aberrant *ISCU* splicing, using an *ISCU* minigene in human RD4 cells. PTBP1 was shown to repress the incorrect splicing while IGF2BP1 and RBM39 enhanced the incorrect splicing [11]. IGF2BP1 is particularly interesting because it shows a higher affinity for the mutant *ISCU* sequence, however, even though it binds RNA it has no known splicing activity. Therefore, how it specifically contributes to enhance the incorrect splicing is not known.

Using a transgenic mouse model, our results confirm that the aberrant splicing of mutant *ISCU* is more pronounced in muscle compared to other tissues, with slow-fiber muscle showing the highest levels of incorrectly spliced mutant *ISCU*. Also, since earlier observations indicate that the expression of the splicing factor serine/arginine-rich splicing factor 3 (SRSF3) is higher in slow-twitch muscles compared to other muscle-types [12, 13], we analyzed the effect of SRSF3 on *ISCU* splicing. Using myoblasts from control and HML patients we could show that overexpression of SRSF3 increase incorrect splicing of *ISCU* while knock-down of SRSF3 decrease incorrect splicing of *ISCU*. SRSF3 may therefore be one of the factors involved in the slow-fiber muscle enhanced mis-splicing.

## Materials and Methods

### Mice

Transgenic mice on a CBA/B6 background were generated using a pCAG vector carrying *ISCU* cDNA for exon 1–5, including the last intron with the HML mutation as well as approximately 1000 bp of the human *ISCU* promoter. Tissues from 9-week-old *ISCU* transgenic mice of both genders were harvested and immediately frozen in liquid nitrogen. Mice were kept in standard cages with free access to water and food (CRM Expanded, SDS). Animals were sacrificed by cervical dislocation. All procedures were approved by the Ethical Committee for Animal Research at Umeå University (A5-12, A74-14).

### Cell lines

Myoblasts from the tibialis anterior of two HML patients (P1, P2) and a healthy control (C) were cultured in 4 volumes of Dulbecco’s modified essential medium (DMEM)(Gibco, Waltham, MA, USA) to 1 volume Medium 199 (Gibco) supplemented with 20% FBS (Gibco), 5 ng/ml recombinant human hepatocyte growth factor (Invitrogen, Waltham, MA, USA) and 50 μg/ml gentamycin. HEK 293T Lenti-X cells (Clontech, Mountain View, CA, USA) were cultured in DMEM (Gibco) supplemented with 1% Glutamax (Gibco), 10% FBS and 1% Pen Strep (Gibco). All cells were grown at 37°C with 5% CO_2_. All chemicals were purchased from Sigma-Aldrich (St. Louis, MO, USA) unless otherwise stated. Studies including humans, where written informed consent was obtained from all participants, were approved by the Regional Ethics Committee for Medical Research at Umeå University (09–105M).

### RNA isolation

RNA was extracted from mouse tissues as described in the RNeasy Fibrous Tissue Mini Kit (Qiagen, Valencia, CA, USA). In brief, mouse tissue samples with a maximum weight of 30 mg were disrupted with a stainless steel bead (5 mm diameter) using TissueLyser LT (Qiagen). Samples were digested with proteinase K, and the supernatant was collected after 3 min centrifugation at 10 000xg and further purified using RNeasy Mini spin columns (Qiagen). RNA was extracted from cells using either the RNeasy Mini Kit (Qiagen) or the NucleoSpin RNA plus kit (Machery-Nagel, Düren, Germany) according to the manufacturers' instructions.

### cDNA synthesis

cDNA was synthesized using the SuperScript^™^ III RT First-Strand Synthesis System for RT-PCR (Invitrogen, Waltham, MA, USA) with random hexamers according to the manufacturer’s instructions. In brief, ~1200 ng of RNA from mouse tissue or ~275 ng of RNA from myoblasts was incubated at 65°C for 5 min with 1 mM dNTP and 50–100 ng random hexamers before the addition of 50 units of SuperScript^™^ III RT enzyme with 10 mM DTT, 5 mM MgCl_2_ in the supplied buffer. Samples were incubated at 25°C for 5 min, 50°C for 1 h followed by 70°C for 15 min.

### Semi-qRTPCR

cDNA was amplified by PCR in ammonium buffer with 1 mM dNTP and 1.25 units TaqPol (Ampliqon III, Odense, Denmark). One forward primer (*ISCU hum/musex3F*, 5’–ATGAAAAGGGGAAGATTGTGG– 3’) was paired with two different reverse primers to amplify the human *ISCU* transgene (*Nifint6R FAM*, 5’–[6FAM] TGCTTGCATGAGAGTCATAAC– 3’) or the endogenous mouse *ISCU* gene (*Musex5RT*.*R*, 5’–[HEX] AAGCAGCTGCTGTGACTG– 3’). The PCR products for the transgene are 418, 504 and 519 bp, where the shortest product represents a correctly spliced transgene *ISCU* RNA and the two longer products represent incorrectly spliced transgene *ISCU* RNA. The PCR was run at 95°C for 1 min followed by 95°C (15 sec), 54°C (20 sec) and 72°C (45 sec) for 30 cycles with a final extension at 72°C for 7 min. The PCR products were analyzed on a 1.2% agarose gel and quantified using a 3730xl DNA analyzer (Applied Biosystems, Waltham, MA, USA). GeneScan 500 LIZ (Applied Biosystems) was used as a size standard. The data were analyzed using the ABI Prism GeneMapper Software Version 3.0 (Applied Biosystems). The proportions of incorrectly and correctly spliced transgene variants were calculated using the peak areas (pa) in the resulting electropherogram. To obtain the relative proportion of incorrectly spliced fragments (% incorrect), each peak area of the two peaks for incorrectly spliced fragments was divided by the total peak area of the three peaks; %incorrect = pa_incorrect_ / (pa_TOTAL_) [14]. All primers were purchased from Sigma-Aldrich (St. Louis, MO, USA). Statistical analyses was performed using Student’s t-test. Number of animals as well as number of experiments performed for each data point are specified in the figure legends.

### qRTPCR

cDNA was amplified by qPCR using SYBR green (Roche, Basel, Switzerland) with a CFX Connect Real-Time PCR Detection System (Bio-Rad, Hercules, CA, USA). The forward primers used to represent correctly and incorrectly spliced endogenous *ISCU* in human myoblasts and the *ISCU* human transgene in mouse tissue were ISCU_ex4_F 5’-ACTGCTCCATGCTGGCTGAA-3’ and ISCU_psex_F 5’- CTGTCGGGTGCTGGCTGAA-3’, respectively. Both forward primers targets exon borders and were paired with the reverse primer Nifint6R 5’-TGCTTGCATGAGAGTCATAAC-3’. An average cycle threshold (Ct) was calculated for each sample triplicate, which was transformed to 2^(-Ct)^ for all further calculations [15, 16]. To calculate the percentage of incorrectly spliced *ISCU* (100 bp), the 2^(-Ct)^ value for the amplicon representing incorrect splicing was divided by the total 2^(-Ct)^ of both *ISCU* amplicons for each sample. The primers for human SRSF3 were SRSF3_F 5’-AGAGCTAGATGGAAGAACACT-3’ and SRSF3_R 5’- ATAATCATCTCGAGGGCGAC-3’, and the primers for the reference gene human β-actin were ACTB_F 5’-GCACAGAGCCTCGCCTT-3’ and ACTB_R 5’-CCTTGCACATGCCGGAG-3’. The primers for mouse SRSF3 were mSRSF3_F 5’-GGTCTCGTAGCCGATCTAGG-3’ and mSRSF3_R 5’-ACCATCTTAAAAATGCACCAAGCT-3’, and the primers for the reference gene mouse GAPDH were mGAPDH_F 5’-TGCCCCCATGTTTGTGATG-3’ and mGAPDH_R 5’-TGTGGTCATGAGCCCTTCC-3’. Data were analyzed using the Bio-Rad CFX Manager software Version 3.1 (Bio-Rad, Hercules, CA, USA). To obtain primer pair efficiencies, standard curves were performed using 4-point, 10-fold dilutions of pooled patient and control myoblast cDNA or mouse cDNA in a representative Ct range. All primers were purchased from Sigma-Aldrich (St. Louis, MO, USA). Statistical analyses was performed using Student’s t-test. Number of animals as well as number of experiments performed for each data point are specified in the figure legends.

### Western blot analysis

Myoblast cells were lysed in a protein lysis buffer (2% SDS, 100 mM Tris-HCl, pH 6.8). Protein concentration was determined by a standard BCA assay (Pierce, Waltham, MA, USA). Denatured samples were separated by SDS-PAGE using Bis-Tris precast gels 4–12% (Bio-Rad, Hercules, CA, USA), and the proteins were transferred to an Amersham Hybond-ECL membrane (GE Healthcare, Fairfield, CT, USA). Membranes were blocked in 5% dry milk in PBST (PBS with 0.1% Tween) for 1 h at room temperature followed by overnight incubation at 4°C with primary antibodies in 0.5% dry milk in PBST. The following primary antibodies were used: rabbit α-SRSF3 (Assay Biotech, Sunnyvale, CA, USA), rabbit α-GAPDH (Cell Signaling, Danvers, MA, USA) and rabbit α-ACTIN (Sigma-Aldrich, St. Louis, MO, USA). The membrane was washed 3x10 min with PBST followed by 1 h incubation with HRP-conjugated α-rabbit antibody (Pierce, Waltham, MA, USA) 1:10 000 in 0.5% dry milk in PBST at room temperature. After 3x10 min washes with PBST, the proteins were visualized using Supersignal West Dura Extended Duration Substrate (Thermo Scientific, Waltham, MA, USA) with Amersham Hyperfilm ECL (GE Healthcare). All chemicals were purchased from Sigma-Aldrich (St. Louis, MO, USA) unless otherwise stated.

### Nuclear extracts

For mouse muscle nuclear extracts, frozen tissues were disrupted using a Mikro-Dismembrator U (Sartorius, Göttingen, Germany) followed by a Dounce homogenizer in Buffer A (10 mM HEPES pH 7.9, 1.5 mM MgCl_2_, 10 mM KCl) with freshly added 1 mM DTT and protease inhibitor cocktail (Complete, Roche, Basel, Switzerland). Myoblasts were pelleted at 300xg for 10 min at 4°C and washed with cold PBS. Cells were resuspended in Buffer A and incubated 15 min on ice. Cell samples were vortexed in pulsed for 10 min and tissue samples were vortexed for 10 seconds in 0.5% NP-40. Nuclei were pelleted at 4500xg for 20 seconds, the cytoplasmic fraction was removed, and nuclei were washed with Buffer C (20 mM HEPES pH 7.9, 1.5 mM MgCl_2_, 420 mM NaCl, 0.2 mM EDTA and 10% glycerol) including protease inhibitors (Complete, Roche) and phosphatase inhibitors (10 mM NaF, 10 mM β-glycerophosphate, 1 mM sodium vanadate). Pellets were resuspended in Buffer C and vigorously shaken on ice for 30 min followed by centrifugation at 14 000xg for 10 min at 4°C. The supernatant, which is the nuclear extract fraction, was collected. Protein concentrations were determined by standard BCA assays (Pierce, Waltham, MA, USA). All chemicals were purchased from Sigma-Aldrich (St. Louis, MO, USA) unless otherwise stated.

### MACS biotinylated molecule isolation

To capture RNA binding factors from nuclear extract, the μMACS Streptavidin kit (MACS Miltenyi Biotec, Bergisch Gladbach, Germany) was used. The 500 μl binding reaction with 500 μg myoblast nuclear extract and 100 pmol oligo in binding buffer (100 mM HEPES pH 8.0, 5 mM EDTA, 50% glycerol, 0.025% Triton X-100 and 250 mM KCl) with 1 mM DTT, 120 ng/μl yeast tRNA, proteinase inhibitors (Complete, Roche, Basel, Switzerland) and 0.04 units/μl RNase OUT (Invitrogen, Waltham, MA, USA) were incubated for 15 min at room temperature. The two *ISCU* oligos used represented normal and mutated *ISCU* (5’–[Biotin] AGCUCCAAUCUUU**C/G**AUUUCAGAAUCUG– 3’), in which the mutated sequence has a C instead of a G. An RNA oligo including a known SRSF3 consensus binding motif was used as a positive control (5’–[Biotin] GCCAUACUCGUCCUCACCAAGUCU– 3’) [17] and a scrambled RNA oligo was used as a negative control (5’–[Biotin] AUCGUGGAUAUAGCAGCGUACUAGUAG– 3’). One hundred microliters streptavidin microbeads (MACS Miltenyi Biotec) was added, followed by 5 min incubation at 4°C. Equilibration buffer (MACS Miltenyi Biotec) followed by 2x500 μl binding buffer was added to the μMACS columns attached to the magnetic MACS multistand (MACS Miltenyi Biotec). The binding reaction was loaded on the column followed by 2x500 μl binding buffer to wash away non-specifically bound proteins. The column was removed from the magnetic field, and the bound nuclear factors along with the beads were eluted with 150 μl PBS. For the Western blot assay, the maximum volume of 33 μl of elute was loaded into each well while 30 μg of nuclear extract was used as a positive control. All chemicals were purchased from Sigma-Aldrich (St. Louis, MO, USA) unless otherwise stated.

### Lentiviral transduction of myoblasts

HEK 293T Lenti-X cells (Clontech, Mountain View, CA, USA) were transfected by calcium phosphate transfection when the cells were 60–70% confluent. Two hours before the transfection, the HEK cells were given media without Pen Strep. For overexpression experiments a total of 80 μg of Lentiviral DNA vectors, (Addgene, Cambridge, MA, USA) pCMV VSVG, pCMV dR8.2 and pCMV R Cre (mCherry) or pLM-SRSF3 (SRSF3 coding sequence cloned into pLM-fSV2A vector), were used in a ratio of 0.7:1:1. For knockdown experiments a total of 55 μg of lentiviral DNA vectors (Addgene, Cambridge, MA, USA), psPAX2 (virus envelope), pMD2.G (virus proteins) and DNA vector (shSRSF3 hairpin sequence with target sequence ACAATGGCAACAAGACGGAAT was cloned into pLKO.1 vector #10878 according to manufacturer’s instructions), were used in a ratio of 1:0.7:1. One-hundred-fifty microliters of 2.5 M CaCl_2_ was added to 1350 μl diluted DNA, and 1500 μl of 2X HEPES (pH 7.00) was then added dropwise while air was bubbled through the mixture. The transfection mixture was kept at room temperature for 30 min and added dropwise to approximately 12x10^6^ HEK cells in a T175 flask. After 4–6 h, the cells were washed with PBS and given fresh media followed by fresh media supplemented with 1 mM sodium butyrate 16 h after the transfection. After 24 h, the cell media were supplemented with 25 mM HEPES, pH 8.0, and after an additional 24 h, the media containing the produced lentiviruses were collected and kept on ice. Cell debris was spun down at 1000xg for 5 min at 4°C, and the supernatant containing the virus was concentrated in an Amicon Ultra-15 100 K centrifugal filter device (Millipore, Billerica, MA, USA), centrifuged at 2000xg at 4°C until the required volume was obtained. Concentrated lentivirus was added to 80 000–100 000 myoblasts in 2 ml media with 10 μg/ml Polybrene (Millipore) in a 6-well plate. For overexpression experiments cells were harvested after 48 h and the expression of mCherry was used as a measure of transfection efficiency. For knockdown experiments the myoblasts were given fresh media with 1.8 μg/ml puromycin 24 h after the transduction and cells were harvested for RNA and protein 72 h after the transduction. All chemicals were purchased from Sigma-Aldrich (St. Louis, MO, USA) unless otherwise stated.

## Results

### Highest levels of incorrectly spliced *ISCU* is observed in mouse skeletal muscle

**Fig 1:**
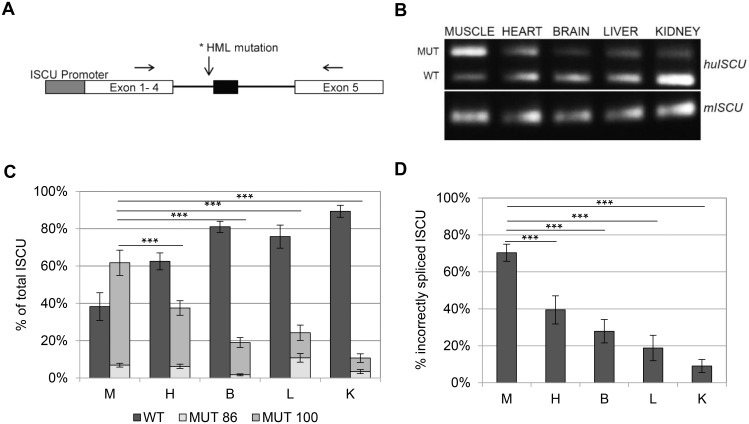
Tissue-specific splicing of the human *ISCU* transgene. A) Schematic representation of the human *ISCU* transgene. Arrows mark the positions of primers used for semi-qRTPCR. The forward primer, present both in human and mouse, is located in exon 3. The reverse primers, specific to either human or mouse, are located in the 5’ UTR sequence in exon 5. The black thick line represents the last intron of the gene, which includes the pseudoexon represented by the black box. B) Semi-qRTPCR performed on cDNA from the human *ISCU* transgene and analyzed on a 1.2% agarose gel. Upper panel, human *ISCU* with incorrect (MUT) and correct (WT) splice variants. Lower panel, mouse endogenous *ISCU*. C) Quantification of the semi-qRTPCR assays using a 3730X DNA fragment analyzer (Applied Biosystems, Waltham, MA, USA). The graph shows the mean relative proportion ± SD of the splice variants, the wildtype (WT) and the two incorrect splice variants in which either 86 (MUT 86) or 100 bp (MUT 100) of intronic sequence (n = 10–14). D) qRTPCR performed on the mouse tissues (n = 6–10). The graph represents relative proportion incorrectly spliced *ISCU* (100bp). Tissues used were; muscle (M), heart (H), brain (B) liver (M) and kidney (K). All semi-qRTPCR and qRTPCR experiments where run in triplicates. (* p < 0.05, ** p < 0.01, *** p < 0.001; Student’s t-test).

The intronic mutation found in the *ISCU* gene of HML patients results in incorrect splicing of the gene where intronic sequence is included in the final mRNA transcript. The incorrect splicing has been shown to occur to a higher extent in muscle compared to heart and liver, which likely explains the muscle-specific phenotype observed in HML [7]. To further investigate the tissue specificity, we generated transgenic mice carrying the human *ISCU* gene with the HML mutation (Fig 1A) and analyzed the splicing of the transgene using semi-qRTPCR. The resulting PCR products represent different splice variants, including the correct splice variant (WT) and the two incorrect splice variants with either 86 (MUT 86) or 100 (MUT 100) bp of intronic sequence (Fig 1B and 1C).

We confirmed the tissue-specific splicing observed in human tissues [7] with most mis-spliced *ISCU* observed in mouse muscle with approximately 62% mis-spliced transcript (Fig 1B and 1C). In the other mouse tissues analyzed, 37% were incorrectly spliced in the heart, 19% in the brain, 24% in the liver and only 10% in the kidney (Fig 1B and 1C). Of the total incorrectly spliced *ISCU*, the 86 bp splice variant was represented by 11% in the liver but only 7, 6, 2 and 3% in the muscle, heart, brain and kidney, respectively (Fig 1C). The data were confirmed by qRTPCR, in which similar levels of incorrectly spliced transcript were observed with 70, 39, 28, 19 and 9% mis-spliced *ISCU* in muscle, heart, brain, liver and kidney, respectively (Fig 1D).

### Slow-twitch muscle shows the highest levels of incorrect splicing of *ISCU*

**Fig 2:**
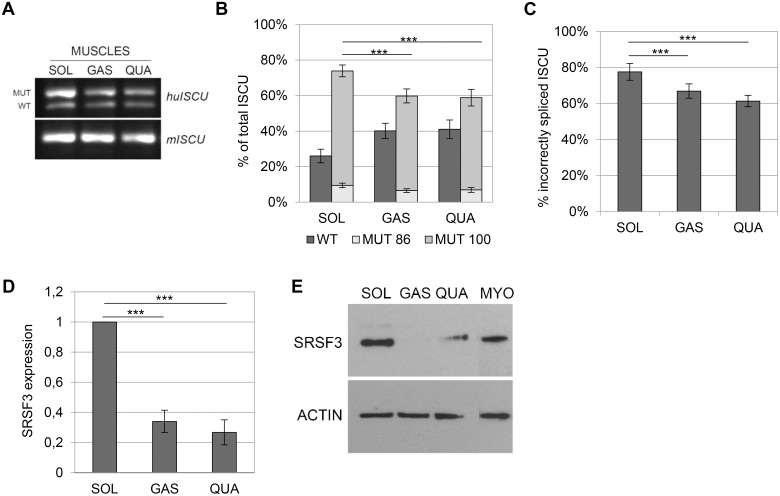
Splicing of the human *ISCU* transgene in mouse muscles. A) Semi- qRTPCR performed on cDNA from the human *ISCU* transgene and analyzed on a 1.2% agarose gel. Tissues used were soleus muscle (SOL), gastrocnemius muscle (GAS) and quadriceps muscle (QUA) B) Quantification of the semi-qRTPCR assays using a 3730X DNA fragment analyzer. The graph shows the mean relative proportion ± SD of the splice variants, WT and the two incorrect splice variants in which either 86 (MUT 86) or 100 bp (MUT 100) (n = 10–14). C) *ISCU* qRTPCR performed on mouse muscles (n = 8). The graph represents mean relative proportion ± SD of incorrectly spliced *ISCU* (100bp). D) SRSF3 qRTPCR using cDNA from mouse muscles (n = 3). The graph represents the mean fold expression ± SD relative to the expression for soleus. GAPDH was used as an internal control. E) Western blot of SRSF3 and ACTIN using nuclear extracts from different mice muscles and protein lysate from control myoblasts (MYO). All semi-qRTPCR and qRTPCR experiments where run in triplicates. (* p < 0.05, ** p < 0.01, *** p < 0.001; Student’s t-test).

The lower levels of incorrectly spliced *ISCU* observed in muscle from transgenic mice compared to muscle from HML patients (~60% vs. ~80%), could be due to the absence of a functional *ISCU* gene copy in the HML patients, but may also be due to differences in the slow-fiber composition of mouse and human muscle, with more fast fibers in mouse muscle. To test the hypothesis that the difference in splicing is dependent on muscle type, we analyzed three different muscles from the human *ISCU* transgene—the soleus, gastrocnemius and quadriceps—which represent different fiber compositions. The soleus consists predominantly of slow-twitch fibers and rely mainly on oxidative metabolism, the gastrocnemius is of a mixed muscle type, and the quadriceps consist predominantly of fast-twitch fibers and rely mainly on glycolysis [18–21]. The levels of incorrectly spliced *ISCU* was 73% in soleus which was significantly higher compared to gastrocnemius (60%) and quadriceps (59%) (Fig 2A and 2B). Again, the semi-qRTPCR results were confirmed by qRTPCR with similar levels of *ISCU* transcript carrying the pseudoexon with 78, 67, and 61% for the soleus, gastrocnemius and quadriceps, respectively (Fig 2C), suggesting that mutant *ISCU* is mis-spliced to a larger extent in muscles consisting of primarily slow fibers.

### SRSF3 enhance the incorrect splicing of *ISCU*

SRSF3 belongs to the conserved family of SR-proteins, which are all well-known splicing factors involved in the regulation of both constitutive and alternative splicing [22–24]. SRSF3 has been shown to be involved in the alternative splicing of several genes, including the regulatory splicing of its own RNA [17, 25–28]. Earlier observations indicate that *SRSF3* expression may be higher in slow-twitch muscles, such as the soleus and anterior latissimus dorsi, compared to other muscles with a mixed or a predominantly fast fiber composition [12, 13]. Because of its role in the regulation of alternatively spliced exons and the muscle-type-specific expression pattern, we speculated that SRSF3 might be involved in the muscle-type-specific splicing of mutant *ISCU*. By qRTPCR and Western blotting, we confirmed that the mouse soleus did indeed express higher levels of *SRSF3* (Fig 2D and 2E). Soleus *SRSF3* mRNA levels were 3 times higher than in the gastrocnemius and 4 times higher than in the quadriceps, and the soleus contained higher levels of SRSF3 protein compared with the gastrocnemius and quadriceps (Fig 2D and 2E).

**Fig 3:**
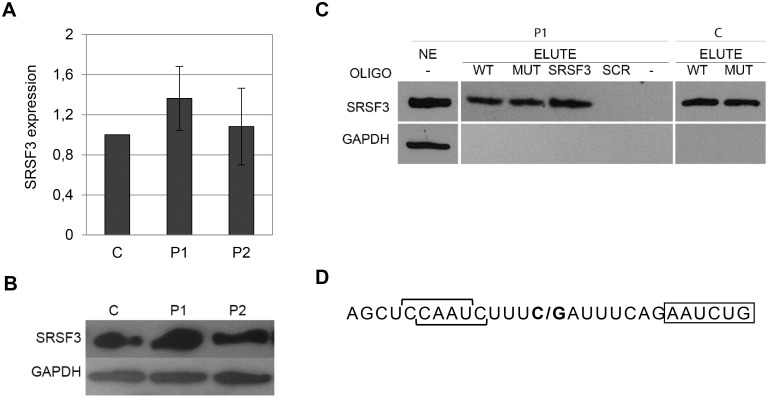
SRSF3 binding to normal and mutated *ISCU* RNA. A) SRSF3 qRTPCR using cDNA from patient (P1, P2) and control (C) myoblasts. The graph presents the mean fold expression ± SD in HML patient samples relative to the control from at least four independent experiments. β-actin was used as an internal control. B) Western blot of SRSF3 and GAPDH in HML patient myoblasts (P1, P2) and a healthy control (C). C) SRSF3 Western blot of the elute fractions (ELUTE) from RNA pull-down assays using patient (P1) and control (C) myoblasts. NE represents input nuclear extract. Nuclear extract was incubated without (-) or with biotinylated RNA oligos; *ISCU* wildtype oligo (WT), *ISCU* mutant oligo (MUT), consensus SRSF3 oligo (SRSF3) or scrambled RNA oligo (SCR). GAPDH was used as a negative control for RNA/protein interaction. The pull-down results were reproduced at least once for each oligo. D) Sequence of the *ISCU* RNA oligos used in the pull-down experiments with proposed SRSF3 binding sites indicated by horizontal square brackets. **C/G** indicates site of HML mutation. Start of the HML pseudoexon is boxed.

To further explore the potential link between SRSF3 and incorrect splicing of *ISCU*, we investigated whether SRSF3 could interact with the *ISCU* RNA transcript. For this, biotinylated *ISCU* RNA oligos, with or without the HML mutation, were used as a bait to fish out RNA binding factors from nuclear extracts prepared from patient (P1) and control (C) myoblasts. qRTPCR and Western blot analysis showed that all three myoblast lines were similar in their *SRSF3* mRNA and protein levels (Fig 3A and 3B). Western blot analysis of captured factors showed that SRSF3 associated with both the normal and mutant RNA oligo to the same extent as to an RNA-oligo with a consensus SRSF3 binding site [17], but not to a scrambled RNA oligo (SCR), indicating a mutation independent interaction between SRSF3 and *ISCU* RNA (Fig 3C). Possible sites of interaction between SRSF3 and the *ISCU* RNA oligos is indicated by horizontal square brackets, based on the proposed consensus motifs by Änkö et al. 2012 [29] (Fig 3D).

**Fig 4:**
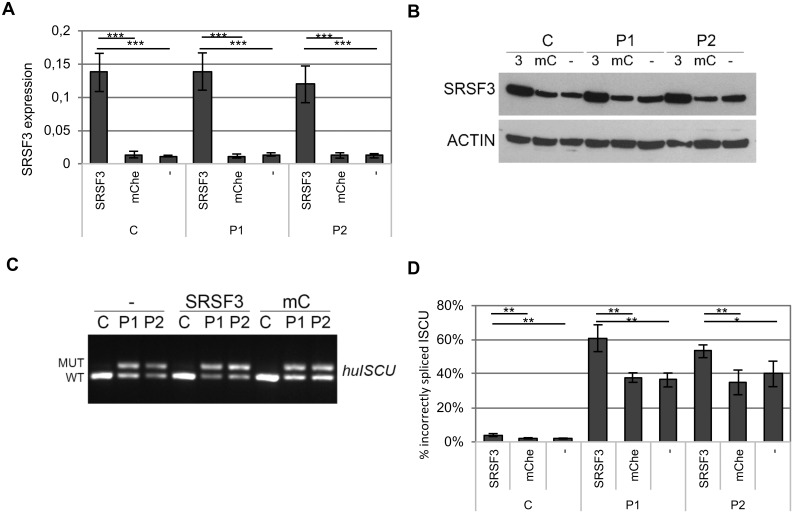
Incorrect splicing of *ISCU* in myoblasts overexpressing SRSF3. A lentivirus-mediated expression vector for either SRSF3 or mCherry (mChe) was introduced into myoblasts from HML patients (P1, P2) and a healthy control (C). A) SRSF3 qRTPCR using cDNA from uninfected myoblasts (-) as well as myoblasts infected with lentivirus-mediated expression vectors, either SRSF3 (SRSF3) or mCherry (mChe). The graph presents the normalized 2^-(ddCt)^ value ± SD for the SRSF3 expression from four independent experiments. β-actin was used as an internal control. B) Western blot showing levels of SRSF3 in myoblasts overexpressing SRSF3 (SRSF3), mCherry (mC) and non-transduced myoblasts (-). ACTIN was used as a loading control. C) Semi-qRTPCR of human *ISCU* with incorrect (MUT) and correct (WT) splice variants from uninfected myoblasts (-), myoblasts infected with lentivirus-mediated vectors expressing SRSF3 (SRSF3) or mCherry (mC). D) Quantification of incorrectly spliced *ISCU* by qRTPCR in uninfected myoblasts (-) as well as myoblasts infected with lentivirus-mediated expression vectors, either SRSF3 (SRSF3) or mCherry (mChe). The graph presents the mean percentage of incorrectly spliced *ISCU* ± SD from four independent experiments (* p < 0.05, ** p < 0.01, *** p < 0.001; Student’s t-test).

To investigate whether SRSF3 could influence the spicing of *ISCU* we overexpressed SRSF in myoblasts from a healthy control (C) and from HML patients (P1, P2) using a lentivirus-mediated SRSF3 expression vector. We observed robust *SRSF3* mRNA and protein overexpression in all three myoblast lines analyzed (Fig 4A and 4B). In untreated myoblasts, with normal levels of SRSF3, approximately 2% of the *ISCU* mRNA was incorrectly spliced in myoblasts from healthy controls compared to approximately 40% in HML patient myoblasts (Fig 4C and 4D). In myoblasts in which *SRSF3* was overexpressed, we observed a significant increase in the level of incorrectly spliced *ISCU*, 1.5 and 1.4-fold in P1 and P2, respectively (Fig 4D), compared to myoblasts transduced by mCherry lentivirus. The incorrect splicing of *ISCU* in the control myoblasts was also increased by as much as 1.7-fold but still remained at a low level (4%) compared to that observed in patient myoblasts (Fig 4D).

**Fig 5:**
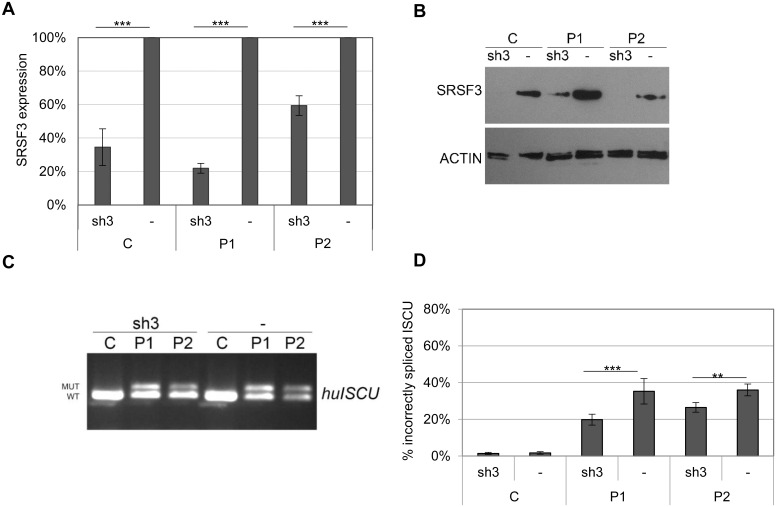
Incorrect splicing of *ISCU* in myoblasts with decreased SRSF3 expression. A lentivirus-mediated expression vector for SRSF3 shRNA (sh3) was introduced into myoblasts from HML patients (P1, P2) and a healthy control (C). A) SRSF3 qRTPCR using cDNA from uninfected myoblasts (-) and myoblasts infected with a shSRSF3 lentivirus-mediated expression vector. The graph present the mean fold change ± SD for the SRSF3 expression from at least three independent experiments. β-actin was used as an internal control. B) Western blot of SRSF3 in non-transduced and transduced myoblasts. ACTIN was used as a loading reference. C) Semi-qRTPCR of human *ISCU* with incorrect (MUT) and correct (WT) splice variants from uninfected myoblasts, (-) or myoblasts infected with lentivirus-mediated vectors expressing shSRSF3 (sh3). D) Quantification of incorrectly spliced *ISCU* by qRTPCR in in non-transduced and transduced myoblasts. The graph presents the mean percentage of incorrectly spliced *ISCU* ± SD from at least three independent experiments (* p < 0.05, ** p < 0.01, *** p < 0.001; Student’s t-test).

In agreement with the SRSF3 overexpression results, knockdown of SRSF3 using a SRSF3 shRNA lentivirus expression vector decreased the mis-splicing of *ISCU* in myoblasts (Fig 5). The SRSF3 RNA expression was knocked down to 20–60% of normal RNA expression and there was also a robust knockdown of SRSF3 protein (Fig 5A and 5B). The SRSF3 knockdown resulted in an approximate 30–50% decrease of incorrectly spliced *ISCU* (Fig 5C and 5D).

## Discussion

We investigated the splicing of mutated *ISCU* in a range of different tissues using a transgenic mouse model expressing human *ISCU* carrying the HML mutation. Using this model, we identified SRSF3 as a splicing factor that may be involved in the muscle-type-specific splicing of *ISCU*. By analyzing the levels of incorrectly spliced *ISCU* in skeletal muscle, heart, brain, liver and kidney in the *ISCU* transgenic mice, we confirmed that muscle contains the highest level of aberrant splicing with 62% incorrectly spliced mutant *ISCU*. In the heart, only 37% of the *ISCU* splicing was incorrect, correlating well with the 30% found in heart from HML patients, whereas in the liver, only 24% of the *ISCU* splice variants were incorrect, which is less than the 46% observed in liver from HML patients. [7]. Brain and kidney, two tissues that have not been previously investigated, showed the lowest levels of aberrant splicing.

As lower levels of incorrectly spliced *ISCU* was observed in muscle from transgenic mice compared to muscle from HML patients (~60% vs. ~80%), we investigated whether there was a difference in splicing in muscles of different fiber types, since muscle from mouse and human differ in fiber composition. The composition of fast and slow muscle fibers defines the functional characteristics of a particular skeletal muscle [18]. Slow-twitch muscle fibers contain large amounts of mitochondria and rely mainly on oxidative metabolism, giving them a high resistance to fatigue [19]. Fast-twitch muscle fibers, however, are more susceptible to fatigue because they rely more on glycolysis [19]. We found that the soleus, a slow-twitch muscle, exhibited the highest level of incorrect splicing compared to the gastrocnemius and quadriceps, which are of a mixed and fast-twitch phenotype, respectively, suggesting a correlation between the repertoire and/or abundance of splicing factors unique to slow-twitch muscles and the incorrect splicing of the *ISCU* transgene. SRSF3 was identified as an interesting candidate for the muscle-type-specific splicing as it has been shown to be expressed at higher levels in the soleus compared to muscles with a different fiber composition [12, 13]. We confirmed this observation, both on an mRNA and protein level, by comparing soleus with the gastrocnemius and quadriceps from our transgenic *ISCU* mice.

SRSF3 is the smallest member of the conserved SR protein family and consists of an N-terminal RNA binding domain and a C-terminal domain rich in arginine and serine [24, 30–32]. SRSF3 is involved in both the general and alternative splicing of a variety of genes, promoting both the exclusion and inclusion of exons, in some cases depending on cell type or as a response to a cellular state [17, 25–28,30, 33]. SRSF3 levels have also been shown to vary in abundance in different tissues and can exert a dose-dependent splicing response resulting in tissue-specific splicing [34, 35]. When overexpressing *SRSF3* in HML patient myoblasts (P1, P2) we found a 1.5 and 1.4 fold increase in the level of incorrectly spliced mutant *ISCU*. The higher levels of SRSF3 resulted in an increase of the *ISCU* transcripts carrying the pseudoexon from approximately 40 to 60% in both patient cell lines, while knockdown of SRSF3 levels in the same cell lines resulted in decreased levels of incorrectly spliced *ISCU*. We could also show that SRSF3 interacts with *ISCU* mRNA in the region of the HML mutation; however, SRSF3 showed no discernable preference for the wildtype or mutated sequence, indicating that the mutation is not critical for SRSF3 binding. This observation is consistent with the 1.7-fold increase in incorrect splicing of *ISCU* in control (C) myoblasts that overexpress SRSF3 but lack the HML mutation.

In summary, using a transgenic mouse model expressing human mutant *ISCU*, we confirmed the muscle-specific splicing of *ISCU* mRNA observed in HML patients. Our transgenic mouse model is therefore a suitable model for the analysis of the *ISCU* splicing pattern and further supports the connection between the muscle-specific splicing of *ISCU* and the muscle-specific symptoms in HML patients. Additionally, we showed that the highest level of incorrect splicing is found in slow-twitch muscle, suggesting a connection between incorrect splicing of *ISCU* and a slow-twitch muscle phenotype. This higher level of incorrect splicing in slow-twitch muscle may be partly regulated by SRSF3, as it is expressed at a higher level in this muscle type. We could also show that SRSF3 can interact, directly or indirectly, with the *ISCU* transcript and that SRSF3 levels affect the levels of mis-spliced *ISCU*. However, since SRSF3 does not differentiate between the normal and mutant sequence, it is most likely not crucial for the mutant specific mis-splicing, but may be one of the factors that regulate the level of *ISCU* mis-splicing in different muscle-types.

## Supporting Information

S1 File Data set file for Figs 1, 2, 4 and 5. (XLSX)
